# Prediction of Tool Eccentricity Effects on the Mechanical Properties of Friction Stir Welded AA5754-H24 Aluminum Alloy Using ANN Model

**DOI:** 10.3390/ma16103777

**Published:** 2023-05-17

**Authors:** Ahmed R. S. Essa, Mohamed M. Z. Ahmed, Aboud R. K. Aboud, Rakan Alyamani, Tamer A. Sebaey

**Affiliations:** 1 Faculty of Engineering, King Salman International University, El-Tor 45615, Egypt; ahmed.ramadan@ksiu.edu.eg; 2 Mechanical Department, Faculty of Technology and Education, Suez University, Suez 43512, Egypt; ab1984oud@gmail.com; 3 Mechanical Engineering Department, College of Engineering at Al Kharj, Prince Sattam bin Abdulaziz University, Al Kharj 11942, Saudi Arabia; 4 Engineering Management Department, College of Engineering, Prince Sultan University, Riyadh 12435, Saudi Arabia; sepaey@hotmail.com; 5 Mechanical Design and Production Department, Faculty of Engineering, Zagazig University, Zagazig 44519, Egypt

**Keywords:** friction stir welding, artificial neural network, tool pin eccentricity, mechanical properties, ANN

## Abstract

The current study uses three different pin eccentricities (e) and six different welding speeds to investigate the impact of pin eccentricity on friction stir welding (FSW) of AA5754-H24. To simulate and forecast the impact of (e) and welding speed on the mechanical properties of friction stir welded joints for (FSWed) AA5754-H24, an artificial neural network (ANN) model was developed. The input parameters for the model in this work are welding speed (WS) and tool pin eccentricity (e). The outputs of the developed ANN model include the mechanical properties of FSW AA5754-H24 (ultimate tensile strength, elongation, hardness of the thermomechanically affected zone (TMAZ), and hardness of the weld nugget zone (NG)). The ANN model yielded a satisfactory performance. The model has been used to predict the mechanical properties of the FSW AA5754 aluminum alloy as a function of TPE and WS with excellent reliability. Experimentally, the tensile strength is increased by increasing both the (e) and the speed, which was already captured from the ANN predictions. The R^2^ values are higher than 0.97 for all the predictions, reflecting the output quality.

## 1. Introduction

As experimentally proven by Thomas et al. [1] in 1991, friction stir welding (FSW) is a solid-state welding process that joins two plates using a non-consumable rotating tool to heat and stir the material plates at the interface of the joint. FSW generates frictional heat through mechanical stirring between the rotating tool and materials to produce strong joints. This process can be utilized for various materials, including aluminum, titanium, steel, and copper alloys, and is known for its ability to produce high-quality defect-free weldments [1]. Friction stir welding (FSW) provides high-integrity joints for welding metal alloys in a solid state. FSW is used in various applications, including shipbuilding, aerospace, automotive, and railways [2,3,4,5,6,7]. For low, medium, and high deformation resistance alloys, investigations have been conducted on how the welding parameters and tool shape affect the quality of the weld [8,9,10,11]. When compared to conventional arc welding methods, FSW offers a number of benefits [12,13,14]. FSW does not experience issues such as thermal distortion, gas porosity brought on by hydrogen absorption during welding, softening of the heat-affected zone, or difficulties in linking sensitivity to solidification cracking, which are particularly frequent in fusion welding processes [15,16,17].

Artificial neural network (ANN) is a technology used in the field of Artificial Intelligence that represents an interconnected network consisting of multi-layered nodes (neurons), including input and output nodes and weighted connections that are meant to simulate a biological neural network [18,19]. Through years of experiments and research, ANN techniques have demonstrated robust learning capabilities and high prediction accuracy through its ability to identify complex mathematical relationships between input and output variables [20]. Accordingly, ANN models have been widely used in the literature as predictive techniques in a range of different fields, including healthcare [21,22], sustainable development [18,19,23], agriculture [23,24], and material science [25,26,27,28].

More specifically, in the field of material science, various ANN models have been used to study friction stir welding. For example, Jamalian et al. [29] used an ANN model to study the joint parameters, mechanical properties, and microstructures of multi-pass FSW of AA5086-H34 joints. Darzi Naghibi et al. [30] also employed an ANN based model to predict the optimal parameters of FSW, including rotary speed, welding speed, and tool offset on maximizing the tensile strength for AA 5052 and AISI 304 joints. Similarly, Buffa et al. [31] developed an ANN model to predict the microhardness values and the microstructure of the FSW of Ti–6Al–4V titanium alloy joints using different process parameters. Another example is presented by D’Orazio et al. [32] where they developed a multi-variable ANN prediction model to predict the vertical force that occurs during the FSW of an AZ31 magnesium alloy sheet. Several other examples exist in the literature of the use of ANN in studying FSW [33,34,35].

In order to predict the impact of pin eccentricity on the mechanical properties of AA 5754-H24 FSW aluminium alloys and to identify the ideal conditions for input parameters to produce FSW joints with good mechanical properties, computer-aided ANN modelling has recently become widely used in the field of friction stir welding [4,31,36,37,38]. Many publications are found in the literature on FSW of aluminum alloys, focusing on the correlation between the friction stir welding parameters and the mechanical properties [31,39]. More recently, research on modern trends involves the effects of tool eccentricity [40,41], tool pin eccentricity [42], and tool pin or shoulder eccentricity in friction stir welding [43,44,45].

Tool eccentricity or tool runout occurs due to the tool offset with respect to the spindle centre. In general, two types of eccentricity can be defined, namely, pin profile eccentricity and tool eccentricity derived from concentric oscillations [43]. Tool pin eccentricity and welding speeds can significantly affect the quality of the friction stir welded joints. Tool pin eccentricity can affect the string action and material flow during the FSW process. Additionally, the material flow behavior around the rotating tool will help achieve high-quality and defect-free joints. Furthermore, the welding speed of the FSW process significantly affects the welded joints’ quality and properties. Due to the selected low welding speed, the heat generated during FSW can accumulate, thereby producing defected joints. On the other hand, when the welding speed is high, the material may not be sufficiently softened, and the material in the stir zone may have insufficient stirring, leading to the production of defected joints. Thus, detecting a suitable welding speed is crucial for conducting defect-free and high-quality weldments using the FSW process [43].

However, there seems to be a lack of research in the literature on the correlation between the tool pin eccentricity and the mechanical properties of FSW of aluminum alloys. Although a few numerical models for the FSW of aluminum alloys can be found in the literature [31,43,46,47], none of them deal with tool pin eccentricity in the process input parameters. This study aims to determine the correlation between welding speed (WS), tool pin eccentricity, and mechanical characteristics of the aluminum alloy FSW AA5754-H24. Neural networks were designed and properly trained based on the experimental data obtained at a tool rotational speed of 600 rpm, different welding speeds, and different values of tool pin eccentricity.

The remainder of this article is structured as follows. Section 2 discusses the data collection methodology for the experimental data used to train the ANN. Section 3 outlines the development and training of the ANN model, as well as the produced results. Section 4 presents a discussion of the produced results from the ANN model. Finally, an overall conclusion of this article is presented in Section 5.

## 2. Collecting the Experimental Data

At Suez University, the friction stir welding of AA5754-H24 aluminum alloy butt joint was carried out using a locally produced FSW machine [48,49,50,51]. The welding samples are produced on two plates that are 5 mm thick, 100 mm broad, and 120 mm long. Table 1 provides the chemical breakdown of the AA5754-H24 aluminium alloys. Table 2 is a list of the measured mechanical characteristics of the employed Al alloys.

**Figure 1:**
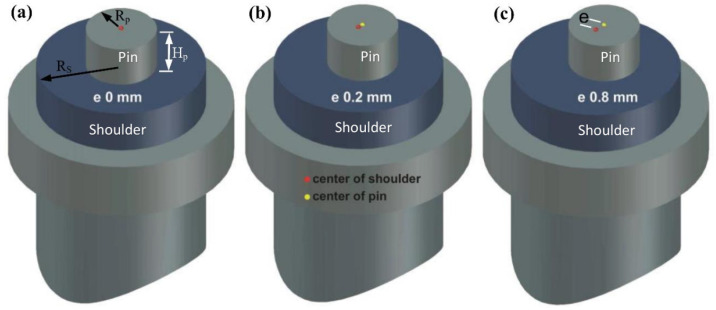
3D CAD drawings of the FSW tool (**a**) e = 0 mm, (**b**) e = 0.2 mm, and (**c**) e = 0.8 mm. Note that e is the value of eccentricity (dimensions in mm).

The welding tool is made of an HRC62 heat-treated cold-worked tool steel rod W302 (0.1% Si, 0.39% C, 5.2% Cr, 0.40% Mn, 1.4% Mo, 0.95% V, and 90.6 wt%). Three tool designs are prepared, as shown in Figure 1. The first is the tool without eccentricity (T0), in which the pin and shoulder axes are aligned. The second tool contains a pin eccentricity of 0.2 mm (T2), i.e., the pin axis is shifted by 0.2 mm from the tool axis. The third tool contains a pin eccentricity of 0.8 mm (T8), i.e., the axis of the designed pin has been shifted 0.8 mm from the axis of the tool. In every instance, a pin that is 4.6 mm long and 6 mm in diameter with 19 mm shoulder diameter and a 2° concavity is employed.

The welding speeds used in FSW are 50, 100, 150, 200, 300, and 500 mm/min. In each case, a rotation speed of 600 rpm is used along with a 0.2 mm tool plunge depth and a 3° tilt angle.

**Figure 2:**
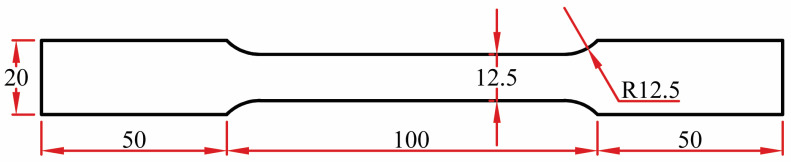
Tensile test specimen. All dimensins are in mm.

By evaluating the joint tensile characteristics and calculating the Vickers hardness in the heat-affected zone (HAZ) and weld nugget zone (WNZ), the FSW joints are evaluated. A “Instron” tensile testing machine with a 300 KN capability is used for tensile testing. Flat tensile specimens with the dimensions specified in Figure 2 are cut perpendicular to the welding direction. The moving head of the machine moves at a velocity of 0.1 mm/s in accordance with ASTM: E8/E8M-16a standard. With a 1 kg load close to the section centerline, the Vickers hardness of the weld nuggets and the thermomechanically affected zone (TMAZ) of the cross sections perpendicular to the welding line direction are measured. To evaluate the mechanical performance of the welding, measurements were made of the ultimate tensile strength, elongation, and hardness of FSWed nugget zone (WNZ) and thermomechanically affected zone (TMAZ). The measured data are shown in Table 3.

For FSW joints made with T0 and T2 tools, it has been found that the tensile strength increases as the welding speed rises. The highest possible tensile strength is reached at 500 mm/min welding speed, and it lowers for the FSW joints made with tool T8 (highest speed). This may be caused by the rate at which heat is applied, which regulates the amount of plastic deformation that occurs. Moreover, a tool pin that is eccentric by more than 0.3 mm produces more heat input [42,52]. This causes a stress concentration, which greatly worsens the mechanical characteristics of the weld joint. Additionally, lowering the rotational speed and increasing the welding speed (by increasing the revolutionary pitch) reduces the heat input. This increases the tensile strength as the welding speed is increased. However, decreasing the welding speed causes more heat input and a low tensile strength [36].

The FSWed nugget zone and the TMAZ are used to measure the hardness values of the FSW welded joints. The junction with the maximum hardness is one that has been welded using a T2 tool with an eccentricity of (e 0.2) at a welding speed of 500 mm/min. The TMAZ is found to have less hardness than the weld nugget zone.

**Figure 3:**
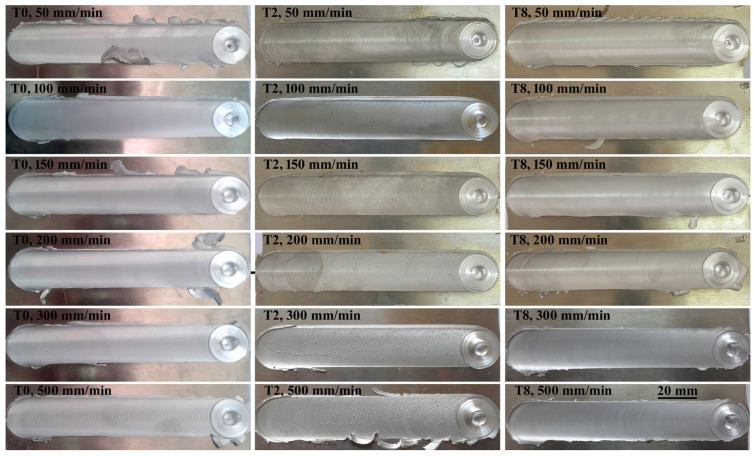
Top surface view of AA5754-H24 FSW joints made using several FSW tools (T0, T2, and T8) and at a rotational speed of 600 rpm.

Figure 3 shows the top surface view of the AA5754-H24 FSW joints at rotating speeds of 600 rpm and various welding speeds of 50, 100, 150, 200, 300, and 500 mm/min using the FSW tools T0, T2, and T8 previously discussed. All joints appear nearly identical on the top surface, which is mostly affected by the shoulder, with the exception that the top surfaces of joints fused at low welding speeds are smoother and have less flash on both the advancing side (AS) and the retreating side (RS). On the other hand, it can be observed that the welding speed has a noticeable impact on the way the surface of welded joints looks. At these high welding speeds, beginning at 300 mm/min, the semicircular banding features visibly grow broader. The measured semicircular banding features spacing agrees with the calculated ones, claims Krishnan [53]. For the welding speeds of 50, 100, 150, 200, 300, and 500 mm/min, the measured semicircular spacing (revolutionary pitch) in this study is 0.083, 0.167, 0.25, 0.333, 0.5, and 0.83 mm, respectively. This spacing appears to be almost identical to the spacing in Figure 3.

## 3. Methodology of the ANN Model

Most manufacturing processes are complex in nature, highly non-linear, and have a large number of input parameters. Currently, no mathematical models can describe the behavior of these processes. Due to the cost-effective and relatively easily understandable nature of ANN models and their ability to be trained using data collected from these complex manufacturing processes, they have been extensively used as predictive techniques [54,55].

**Figure 4:**
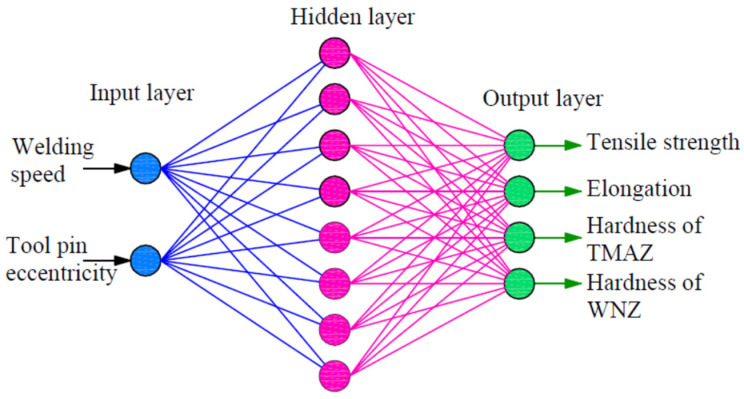
The structure of the ANN model used in this work.

The input layer, the hidden layer, and the output layer are the three layers that constitute an ANN. All of the input parameters are contained in the input layer. The hidden layer processes data from the input layer, after which the final (output) layer computes the next output vector. In Figure 4, the three layers of the ANN model utilized in this study are shown schematically.

An ANN consists of simple synchronous processing elements that are inspired by the biological nervous system. The basic unit in the ANN is the neuron. Neurons are connected by links known as synapses, and associated with each synapse is a weight factor. In this work, two training algorithms (gradient descent with momentum algorithm, and Levenberg–Marquardt Algorithm) were used with a single hidden layer (eight neurons). The inputs and outputs were normalized in the range of 0:1. Thus, the network architecture consisted of two input neurons, eight hidden neurons with a nonlinear activation function, a logistic sigmoid (logsig), and four output neurons with a linear activation function. Table 4 shows the trained network weights and biases.

The ANN is trained and tested using MATLAB. After training the ANN successfully, it is tested using measured data that are different from what was used in the training processes. The number of neurons is increased through the training process from five to nine to define the output accurately.

To train and test an ANN neural network, we need ANN training (patterns), input data, and corresponding target values (measured data). A total of 18 patterns have been obtained in this work from the experiments. The welding speed and tool pin eccentricity are inputs to the network, while the outputs include tensile strength, elongation, TMAZ hardness, and weld nugget zone hardness. As a result, the architecture of the ANN changes to 2-8-4, where 2 represents the input values, 8 represents the number of hidden layer neurons, and 4 represents the outputs. Four measured results have been utilized as test data, and fourteen out of a total of eighteen measured results have been used as data sets to train the network (75% training data against 25% test data). The measured results are used to develop and test the ANN model.

## 4. Results and Discussion

**Figure 5:**
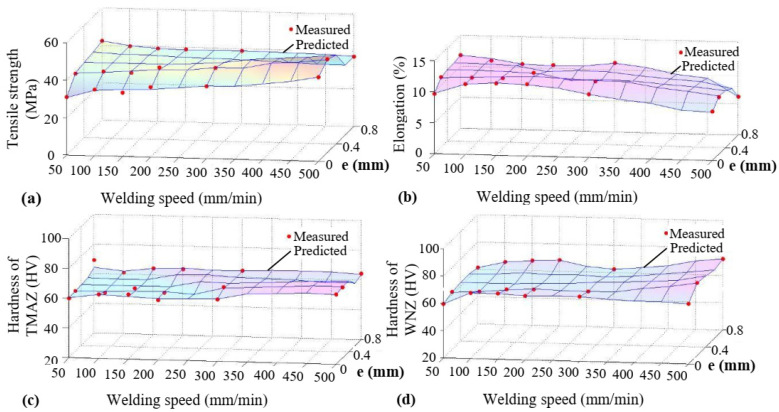
Variation in predicted and measured data. (**a**) tensile strength; (**b**) elongation; (**c**) hardness of TMAZ; (**d**) hardness of weld nugget.

Figure 5 shows the variation in the mechanical properties of AA5754-H24 FSW joints with tool pin eccentricity and welding speed, and the predicted new data obtained from the network after the training process. As can be seen from the figure, the predicted values match very well with the measured data.

**Figure 6:**
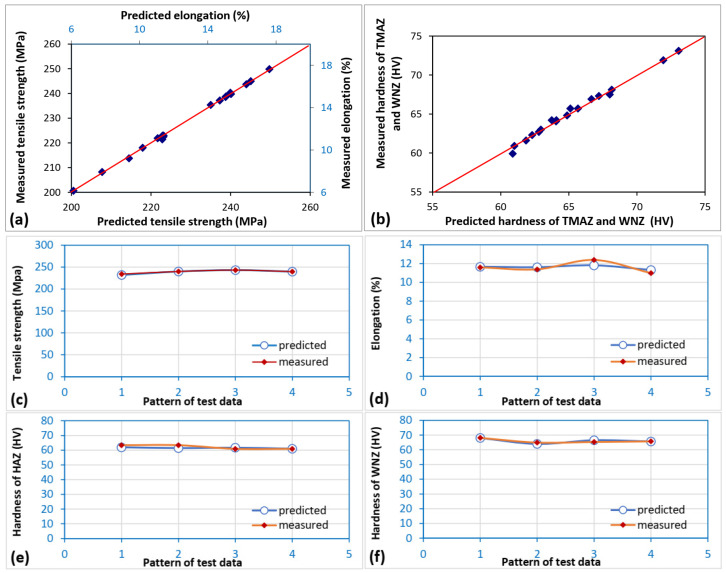
Comparison of measured and predicted data for (**a**,**b**) training data, (**c**) test data of tensile strength, (**d**) test data of elongation, (**e**) test data of hardness of TMAZ, and (**f**) test data of hardness of weld nugget.

The results of the training and testing of the ANN model for the measured and predicted data are given in Figure 6a,b. The results show that both the measured and predicted data sets are highly similar, with variance values (R) of 0.9986 and 0.9889 for training and testing, respectively. Figure 6c–f compare the measured and predicted results of the test data. From these figures, it can be observed that the measured and predicted values are also highly similar. The ability of a neural network to reliably anticipate the results of the test data that have not yet been seen is, nevertheless, its main criterion for quality.

The arithmetical mean of the number of inputs and outputs is typically used to determine how many neurons should be employed in the hidden layer. As a result, it is advised to employ a scaled conjugate gradient (SCG) with eight hidden layer neurons in the current application. Five to nine hidden layers are used in this application’s testing. Based on these outcomes, the model performed well. The network with nine hidden layer neurons has produced the same accuracy of results for the eight hidden layer neurons. Accordingly, only eight hidden layer neurons have been used in the present application.

The prediction accuracy for the results produced by the network is based on statistical methods. Errors occurring during the training and testing processes are called the root-mean-squared (RMS), an absolute fraction of variance (R2), and mean error percentage values. These are calculated as follows [14]:

(1) $$RMS={\left(\left(1/p\right)\sum {\left|tj-oj\right|}^{2}\right)}^{1/2},\;R^{2}=1-\left(\frac{\sum_{j}{\left(tj-oj\right)}^{2}}{\sum_{j}{\left(oj\right)}^{2}}\right),$$

(2) $$mean\;\%\;error=\frac{1}{p}\sum_{j}\left(\frac{tj-oj}{tj}\times 100\right)$$

The results are shown in Table 5.

As can be seen in Table 5, the absolute fraction of variance (R2) values for the outputs of the tensile strength, elongation, hardness of TMAZ and weld metal for the training data are all greater than 0.99 and for the test data are all greater than 0.98, except for the hardness of the TMAZ, which is 0.97, respectively. Based on these results, it can be concluded that the proposed model is highly accurate.

## 5. Conclusions

In this study, AA5754-H24 has been friction-welded using a wide range of welding speeds at three different tool pin eccentricities, and an ANN model has been developed to predict mechanical properties. The experimental results have been used to train the ANN model used afterward for prediction. Based on the obtained results, the following conclusions can be outlined:

- The ANN model has been developed based on the FSW experimental work data of AA5754-H24, in which FS was welded using 0, 0.2, and 0.8 mm TPE and welding speeds of 50, 100, 150, 200, 300, and 500 mm/min.
- The ANN model was successfully used to predict the effect of tool pin eccentricity on the mechanical properties of FSW AA 5547-H24, and the networks can be used as an alternative.
- The RMS error values for the ultimate tensile strength, elongation, hardness of the TMAZ, and weld metal for the test data were 1.1346, 0.3515, 1.2759, and 0.3743, respectively; the R2 values are all greater than 0.98, except for the hardness of the TMAZ, which is 0.97.
- It is found that the correlations between the measured and predicted values of the ultimate tensile strength, elongation, and hardness of the weld metal are better than those of the hardness of the TMAZ.

## Figures and Tables

**Table 1:** Chemical composition of aluminium alloy AA5754.

wt.%								
Fe	Si	Mn	Cu	Mg	Zn	Ti	Cr	Al
0.40 ± 0.03	0.40 ± 0.02	0.50 ± 0.04	0.10 ± 0.01	2.6–3.2 ± 0.03	0.20 ± 0.01	0.15 ± 0.01	0.30 ± 0.02	rest -

**Table 2:** Measured mechanical characteristics of AA5754.

Proof Stress 0.2% (MPa)	Tensile Strength (MPa)	Elongation (%)
205 ± 5	260.57 ± 4.60	12.93 ± 1.80

**Table 3:** Measured data used to train the ANN.

No.	e (mm)	Welding Speed (mm/min)	Tensile Strength (Mpa)	Elongation (%)	Average Hardness (HV)	
					HAZ	WNZ
1	0	50	231.00	9.66	59.9 ± 0.3	59.5 ± 0.6
2	0	100	235.38	11.36	63.0 ± 0.1	68.1 ± 0.9
3	0	150	234.06	11.60	63.4 ± 0.1	68.1 ± 0.3
4	0	200	237.43	11.58	60.2 ± 0.2	66.9 ± 0.7
5	0	300	238.58	10.21	61.6 ± 0.4	67.3 ± 0.3
6	0	500	244.95	7.92	66.9 ± 0.5	64.05 ± 0.5
7	0.2	50	238.80	11.32	60.6 ± 0.7	62.0 ± 0.6
8	0.2	100	240.31	11.35	59.9 ± 0.3	62.7 ± 0.4
9	0.2	150	240.02	11.39	63.4 ± 0.4	64.9 ± 0.8
10	0.2	200	243.22	12.39	60.9 ± 0.8	65.3 ± 0.3
11	0.2	300	243.69	11.25	65.7 ± 0.4	64.82 ± 0.7
12	0.2	500	249.87	9.21	67.5 ± 0.6	73.1 ± 0.6
13	0.8	50	242.26	11.74	68.9 ± 0.9	61.1 ± 0.9
14	0.8	100	239.76	10.99	60.9 ± 0.6	65.7 ± 0.4
15	0.8	150	238.97	10.54	64.2 ± 0.8	67.2 ± 0.7
16	0.8	200	238.87	10.50	66.9 ± 0.5	68.1 ± 0.8
17	0.8	300	238.74	11.10	58.9 ± 0.3	62.3 ± 0.3
18	0.8	500	237.15	6.14	64.2 ± 0.4	71.9 ± 0.5

**Table 4:** Weights and biases for the ANN after the training stage.

**Layer**	**Neurons per Layer**		**Weights**								**Biases**
		i	W_i1_	W_i2_							b_i_
1	8	1	2.7009	4.5649							−1.7555
		2	−2.0315	−3.4643							1.3986
		3	−3.1972	18.3450							22.1916
		4	3.9171	−3.6640							1.4856
		5	−15.2546	−39.2993							−7.1898
		6	3.8393	−3.3341							1.5511
		7	7.4015	1.4190							6.2523
		8	0.6582	−1.6493							1.0211
		j	W_j1_	W_j2_	W_j3_	W_j4_	W_j5_	W_j6_	W_j7_	W_j8_	b_j_
2	4	1	0.6098	0.6742	−0.5132	4.8089	0.0594	−5.3504	0.7177	−0.0521	0.2423
		2	3.0641	3.2349	−0.1842	5.0439	0.0677	−5.7714	0.5053	1.1842	−0.2822
		3	−2.0024	−2.1078	−4.1730	9.0617	−0.4064	−9.6011	0.4018	0.1366	3.8853
		4	−17.6851	−19.4517	0.8332	15.4643	0.4267	−16.9510	0.8747	0.9753	−0.3709

**Table 5:** Statistical values of predication data.

	RMS of Train	R^2^ of Train	Mean Error of Train	RMS of Test	R^2^ of Test	Mean Error of Test
Tensile strength	0.3993	0.9993	0.0608	1.1346	0.9946	0.2512
Elongation	0.0904	0.9992	0.0536	0.3515	0.9894	0.2865
Hardness of TMAZ	0.5167	0.9958	0.3159	1.2759	0.9735	3.3267
Hardness of WNZ	0.0755	0.9999	0.0036	0.3743	0.9979	0.0101

## Data Availability

Data will be available upon request from the corresponding authors.

## Nomenclature

bi	Biases of layer one, i = 1, 2, 3, …, 8
bj	Biases of layer two, j = 1, 2, 3, 4
oj	Output (prediction data), where j = 1, 2, 3, …, 14 for training and j = 1, 2, 3, 4 for testing.
*p*	Samples (*p* = 14 for training, *p* = 4 for testing).
R2	Absolute fraction of variance
RMS	Root-mean squared
tj	Target (measured data), where j = 1, 2, 3, …, 14 for training and j = 1, 2, 3, 4 for testing.
TMAZ	Thermo mechanical affected zone
Wi	Weights of layer one, i = 1, 2, 3, …, 8
Wj	Weights of layer two, j = 1, 2, 3, 4
WNZ	Weld nugget zone
